# A UK Single-Center, Retrospective, Noninterventional Study of Clinical Outcomes and Costs of Two BotulinumtoxinA Treatments for Limb Spasticity

**DOI:** 10.3390/toxins15090532

**Published:** 2023-08-30

**Authors:** Clive Bezzina, Vadim Degtiar, Natalya Danchenko, Pascal Maisonobe, Benjamin Davis, Emanuel Engmann, Elodie Guyon, Sophie Lecanuet, John Whalen

**Affiliations:** 1 North Staffordshire Rehabilitation Centre, Haywood Hospital, Stoke-on-Trent ST6 7AG, UK; 2 Ipsen, Slough SL1 3XE, UK; 3 Ipsen, 92100 Boulogne-Billancourt, France

**Keywords:** adult limb spasticity, stroke, botulinumtoxinA, abobotulinumtoxinA, aboBoNT-A, clinical practice, healthcare cost, onabotulinumtoxinA, onaBoNT-A, rehabilitation

## Abstract

Service model changes at the North Staffordshire Rehabilitation Centre (UK) included switching spasticity treatment from onabotulinumtoxinA (onaBoNT-A) to abobotulinumtoxinA (aboBoNT-A). This noninterventional, retrospective, longitudinal study (NCT04396704) describes the clinical and economic outcomes in toxin-naive adults with spasticity who received onaBoNT-A (Cohort 1; 2015–2017) or aboBoNT-A (Cohort 2; 2017–2019). Outcomes included Goal Attainment Scale T (GAS-T) score, treatment satisfaction, quality of life (QoL; EQ-5D visual analog scale [VAS] score), and treatment costs. Adverse events were recorded for Cohort 2. Cohort 1 included 60 patients (mean [standard deviation] dose, 206.0 [98.8] U); Cohort 2 included 54 patients (753.7 [457.3] U). Mean (95% confidence interval) GAS-T scores for Cohorts 1 and 2 were 43.1 (39.3–46.9) and 47.8 (43.7–51.9) at Week 6, and 43.2 and 44.3 at Week 12, respectively. In both cohorts most patients were satisfied with treatment. At Week 12, QoL had not changed in Cohort 1 but had improved in Cohort 2 (EQ-5D VAS, −5). Mean estimated per-patient costs (in 2021) for Cohorts 1 and 2 were £315.56 and £249.25, respectively, at Week 6, and £343.20 and £273.21, respectively, at Week 12. Fifteen non–treatment-related serious adverse events and two deaths were recorded. These data may warrant a larger prospective study powered to compare outcomes of aboBoNT-A and onaBoNT-A.

## Plain Language Summary

After a stroke, up to 40% of patients might experience limb stiffness (limb spasticity) that leads to difficulties in moving and performing everyday tasks. To ensure best standard of care for patients with limb spasticity, the various clinicians and therapists involved in a patient’s care (e.g., neurologists, physiotherapists, occupational therapists) need to collaborate in one team called “a multidisciplinary team”. Often, effective management of limb spasticity combines physiotherapy with botulinum toxin injections. The aim of this study from one hospital in England was to describe the achievement of patient treatment goals and treatment costs following a practice change in managing limb spasticity. This change included creating a multidisciplinary team and switching treatment from one botulinum toxin to another. This study looked at past records of patients who were prescribed spasticity treatment by their leading clinicians. Patients were adults with upper and/or lower limb spasticity who had not received botulinum toxin injections before. Sixty patients received onabotulinumtoxinA between 1 March 2015 and 29 May 2017, and 54 patients received abobotulinumtoxinA between 30 May 2017 and 30 March 2019. We measured how effective the treatments were and treatment costs at 6 and 12 weeks following the injection for all patients. Adverse events, which are any undesired effects that a patient may have after receiving medical treatment, were monitored only for patients who received abobotulinumtoxinA. The findings from this study suggest that a multidisciplinary approach and change in toxin may have a beneficial effect on achieving treatment goals, patient satisfaction, and quality of life, and may decrease treatment costs. AbobotulinumtoxinA was overall well-tolerated, and no adverse events were linked to this treatment. Further studies in patients with limb spasticity are needed to compare how effective abobotulinumtoxinA and onabotulinumtoxinA treatments are and their costs.

## 1. Introduction

Spasticity is a burdensome sensorimotor disorder resulting from an upper motor neuron lesion, which presents as intermittent or sustained involuntary activation of muscles [1]. Spasticity is commonly associated with several neurological conditions, but the prevalence of limb spasticity (LS) varies, developing in approximately 40% of patients with stroke and 80% of patients with multiple sclerosis [2,3]. LS is associated with pain and disability and can negatively affect patients’ autonomy and various aspects of their quality of life (QoL), including their ability to work, socialize, and perform daily tasks [2,3].

The goals of LS management are to improve patients’ QoL, to ameliorate symptoms, and to improve function of the affected limb(s) [3]. Clinical guidelines recommend that LS management is based primarily on physiotherapy, including postural management, exercise, stretching and strengthening of limbs, splinting, and pain relief [4]. When physiotherapy is insufficient for patients, pharmacological intervention may be necessary [3].

There are barriers to appropriate referral and treatment for patients with spasticity. Spasticity is often under-recognized, and there is no patient pathway clearly identified in the UK [3,5,6]. In the literature, it is recommended that an effective spasticity management plan is based on an approach that combines assessment by a multidisciplinary team with specialist knowledge of spasticity, pharmacological intervention, and physical rehabilitation [3,5,6,7]. Furthermore, establishing an appropriate patient pathway for spasticity management, as well as early diagnosis and intervention, may help patient rehabilitation and prevent long-term complications [3,5,6,7].

In many cases, an effective approach for the treatment of LS combines physiotherapy with intramuscular injections of botulinumtoxinA (BoNT-A) [3,4,8]. A noticeable clinical improvement in spasticity is often achieved following the first BoNT-A injection, with response rates of approximately 70% [8,9,10]. At the time of this study, among available BoNT-A treatments, the Royal College of Physicians and American Academy of Neurology recommend abobotulinumtoxinA (aboBoNT-A) and onabotulinumtoxinA (onaBoNT-A) for the management of both upper and lower LS, and incobotulinumtoxinA for treatment of upper LS [3,11,12,13,14].

To improve symptom control when response to BoNT-A treatment is insufficient or when spasticity reemerges, patients might need repeated injections in line with the product specifications [3].

A recent longitudinal, real-world study including 953 patients showed that aboBoNT-A treatment for upper LS is associated with longer injection intervals than other BoNT-A products [9]. Additionally, results from previous pharmacoeconomic studies in patients with LS [15,16,17] or cervical dystonia [18] show that aboBoNT-A treatment resulted in better QoL than onaBoNT-A treatment [16,17], and that it had a favorable cost profile compared with onaBoNT-A treatment, with the potential for cost savings without a reduction in clinical effectiveness [15,16,17,18]. This may support a switch from onaBoNT-A to aboBoNT-A in some cases to deliver more efficient management of spasticity in the UK [3,17,19,20].

To date, aboBoNT-A and onaBoNT-A have been compared in clinical trial settings only for the treatment of forehead lines and facial synkinesis [21,22]. To our knowledge, no studies have directly compared clinical outcomes of LS treatment with aboBoNT-A and onaBoNT-A at a single center. It would be of interest to describe the impact on treatment outcomes of changing the toxin formulation in routine clinical practice from onaBoNT-A to aboBoNT-A. As with any change in clinical practice, it is also important to understand the economic implications.

In 2015, changes to the service model were implemented at the North Staffordshire Rehabilitation Centre in the UK. The service redesign included a change of the toxin used for the treatment of LS, from onaBoNT-A to aboBoNT-A, which was introduced in 2017. This allowed for a service evaluation and a retrospective study to examine the impact of practice change on clinical outcomes and costs.

The aim of this retrospective, single-center study was to describe the real-world clinical and economic outcomes of practice change that included switching treatment from onaBoNT-A to aboBoNT-A in toxin-naive adult patients with upper and/or lower LS in the UK.

## 2. Results

### 2.1. Baseline Demographics and Clinical Characteristics

In total, 114 patients were included in the full analysis set: 60 in Cohort 1, who received onaBoNT-A, and 54 in Cohort 2, who received aboBoNT-A. The safety analysis set comprised data from Cohort 2 only; no safety data were collected for Cohort 1 because this was out of the scope of the study objectives.

Baseline demographics and clinical characteristics are summarized in Table 1. Overall, demographics and clinical characteristics were similar between cohorts, although there were some differences in underlying neurological condition and baseline pain medication use.

### 2.2. Description of Injection Practice

The injection characteristics at the first BoNT-A administration are presented in Table S1 of the supplementary materials. Across both cohorts, more than 50% of the patients received injections in the lower limbs only, approximately 40% were injected in the upper limbs only, and fewer than 10% received injections in both the upper and lower limbs. Injected muscles were similar across the cohorts for the first BoNT-A injection (Table S2). For Cohort 2, the most used guidance technique at first injection was ultrasound (Table S3); this information was not routinely collected for Cohort 1.

The mean (standard deviation [SD]) total dose per patient at the first injection was 206.0 (98.8) units (U) in Cohort 1 (*N* = 60) and 753.7 (457.3) U in Cohort 2 (*N* = 54) (Table S1). The mean (SD) total dose per patient for reinjections was 236.4 (80.8) U for patients in Cohort 1 (*n* = 14) and 703.3 (427.5) U for patients in Cohort 2 (*n* = 30) (Table 2). Note that aboBoNT-A and onaBoNT-A units are noninterchangeable.

### 2.3. Treatment Outcomes

#### 2.3.1. Goal Attainment Scale T Scores

**Figure 1:**
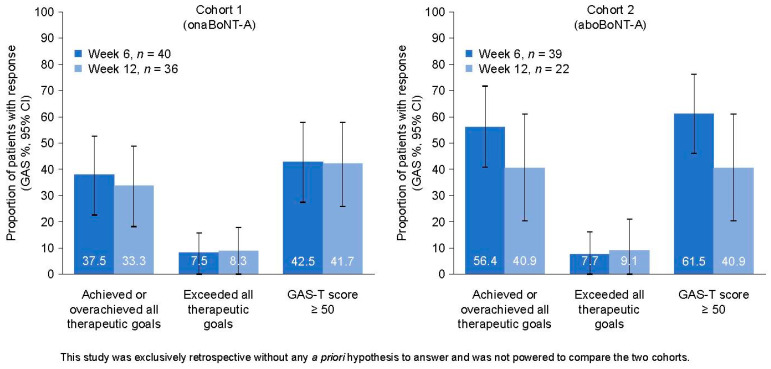
Clinical outcomes of BoNT-A treatment in toxin-naive adults with LS. Response is defined as achieving or overachieving all therapeutic goals (GAS); data are shown as proportions of patients with response in each category, with vertical lines representing the 95% CIs. The *n* number corresponds to the number of patients with available data (see Table S4). AboBoNT-A, abobotulinumtoxinA; BoNT-A, botulinumtoxinA; CI, confidence interval; GAS, Goal Attainment Scale; GAS-T, Goal Attainment Scale T; LS, limb spasticity; onaBoNT-A, onabotulinumtoxinA.

The baseline Goal Attainment Scale T (GAS-T) score for every patient was −2, and changes in score were assessed at Week 6 and Week 12. In the primary outcome analysis (Figure 1; Table S4), mean (95% confidence interval [CI]) GAS-T scores at Week 6 were 43.1 (39.3–46.9) and 47.8 (43.7–51.9) for Cohort 1 (*n* = 40) and Cohort 2 (*n* = 39), respectively. At Week 12, mean (95% CI) GAS-T scores were 43.2 (38.5–47.9) and 44.3 (39.0–49.7) for Cohort 1 (*n* = 36) and Cohort 2 (*n* = 22), respectively.

The proportions (95% CI) of patients who achieved or overachieved all therapeutic goals at Week 6 were 37.5% (22.5–52.5) and 56.4% (40.8–72.0) in Cohort 1 (*n* = 40) and Cohort 2 (*n* = 39), respectively (Table S4). At Week 12, these proportions (95% CI) were 33.3% (17.9–48.7) and 40.9% (20.4–61.5) in Cohort 1 (*n* = 36) and Cohort 2 (*n* = 22), respectively.

In an exploratory statistical comparison of the GAS-T scores, no statistically significant differences between the groups were found at Week 6 or Week 12 (Table S4). In an exploratory statistical comparison of the proportions of patients who achieved prespecified therapeutic goals, there were no significant differences found between treatment groups, although there was a numerical difference in favor of Cohort 2 over Cohort 1.

#### 2.3.2. Reinjections

Patients were reviewed by their physician at Week 6 and Week 12 as per routine practice. As expected, no patients in either cohort were reinjected at Week 6 (Table 2). At Week 12, five (8.3%) of the 60 patients in Cohort 1 and seven (13.0%) of the 54 patients in Cohort 2 required reinjection. During the 24-week follow-up period, the time between the date of first BoNT-A injection and reinjection was similar for patients in Cohort 1 and those in Cohort 2 (mean [SD]: 16.4 [2.7] weeks for Cohort 1 [*n* = 16] and 17.4 [3.7] weeks for Cohort 2 [*n* = 22]) (Table 2).

#### 2.3.3. Patient-Reported Outcomes

The impact of treatment on QoL was determined using the EQ-5D visual analog scale (VAS) score. Overall, there was no change in EQ-5D VAS score between baseline and Week 6 in either group (median [first quartile, third quartile] change: 0 [−7.5, 0.0] for Cohort 1 [*n* = 35] and 0 [−5.0, 0.0] for Cohort 2 [*n* = 24]) (Table 3). At Week 12, QoL had improved in Cohort 2, and there was no change in QoL in Cohort 1 (median [first quartile, third quartile] change from baseline in EQ-VAS score: −5.0 [−28.8, 0.0] for Cohort 2 [*n* = 14] and 0 [−10.0, 0.0] for Cohort 1 [*n* = 24]) (Table 3).

**Figure 2:**
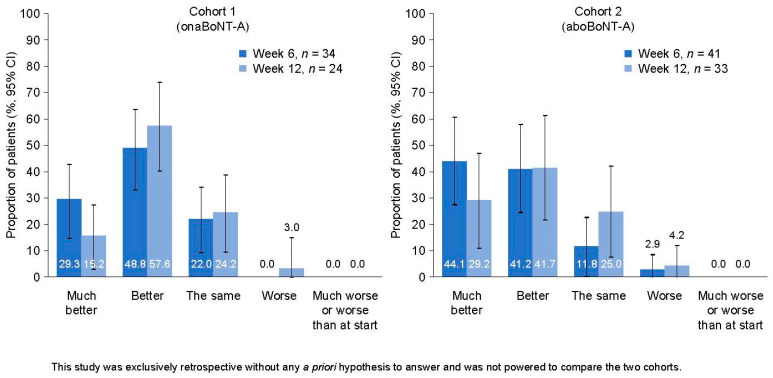
BoNT-A treatment satisfaction in toxin-naive adults with LS. Descriptive analysis (compared with baseline) of patient-reported response for treatment satisfaction using a five-category Likert scale questionnaire ranging from ‘worse than at start’ to ‘much better’; data are shown as proportions of patients in each category, with vertical lines representing the 95% CIs. The *n* number corresponds to the number of patients with available data. AboBoNT-A, abobotulinumtoxinA; BoNT-A, botulinumtoxinA; CI, confidence interval; LS, limb spasticity; onaBoNT-A, onabotulinumtoxinA.

Patients were asked about their level of satisfaction with treatment at Week 6 and Week 12 compared with baseline. Most patients in each cohort rated their treatment satisfaction as ‘better’ or ‘much better’ at both time points (Figure 2).

The proportion of patients who reported ‘much better’ satisfaction at Week 6 than at baseline was greater in Cohort 2 (44.1%, *n* = 34) than in Cohort 1 (29.3%, *n* = 41). Similarly, the proportion of patients who reported ‘much better’ satisfaction at Week 12 than at baseline was greater in Cohort 2 (29.2%, *n* = 24) than in Cohort 1 (15.2%, *n* = 33) (Figure 2). One patient in Cohort 2 at Week 6 and two patients (one in each cohort) at Week 12 rated their satisfaction with treatment as ‘worse’ than at baseline.

### 2.4. Cost of BoNT-A Treatment

There was no increase in treatment costs between the index date and Week 6 because no patient was reinjected in either group (Table 4). The mean estimated costs (in 2021) per patient at Week 6 were £315.56 for Cohort 1 and £249.25 for Cohort 2, respectively, and increased at Week 12 as a result of reinjection to £343.20 and £273.21, respectively (Table 4). For patients who experienced a response to treatment, the cost per responder at Week 6 was £276.40 for Cohort 1 and £233.80 for Cohort 2, respectively, and at Week 12 were £391.57 and £290.89, respectively (Table 4).

Overall, estimated treatment costs per patient at Week 6 and Week 12 were numerically lower in Cohort 2 than in Cohort 1 (Table 4).

### 2.5. Safety

Safety data were collected only for patients in Cohort 2 (Table S5). During the study period, 15 adverse events (AEs) were reported in eight patients (14.8%); all were serious AEs (SAEs) and severe in intensity. The most frequently reported SAEs included nervous system disorders (*n* = 3; 5.6%), and infections and infestations (*n* = 3; 5.6%). Two patients (3.7%) had SAEs leading to death: one had seizures and Parkinson’s disease progression; the other patient’s refusal and inability to take oral medication resulted in death. None of the SAEs were assessed as related to aboBoNT-A treatment. One patient had three AEs with ‘relatedness not known’.

There were no treatment discontinuations due to AEs. Special situations (off-label muscle injections, inappropriate schedule of product administration, lack of therapeutic effectiveness, and supramaximal dose) were reported in 21 patients (38.9%). Twenty-one patients (38.9%) received off-label muscle injections, inappropriate schedule of product administration was reported in one patient (1.9%) and lack of therapeutic effectiveness was reported in one patient (1.9%). Five patients (9.3%) received aboBoNT-A injections in the upper limbs of which the total dose across multiple muscles exceeded the maximum authorized dose stated in the summary of product characteristics (supramaximal dose).

### 2.6. Service Redesign

The positive outcomes of the service redesign included reduced waiting times for patients, who were assessed by a multidisciplinary team earlier in the patient pathway. Following the reconfiguration of the service, referred patients were assessed upfront by a doctor and physiotherapist, which allowed direct triaging of eligible patients into the spasticity clinic. The joint upfront assessment averted the (former) 8–12-week wait-time between initial physician assessment and subsequent spasticity clinic visit and therapy assessments. Rather than requiring additional resources within the care service, the remodeled pathway utilized the existing physiotherapy resource more efficiently (earlier in the pathway). This ensures more timely input of therapist expertise, thereby reducing spasticity clinic waiting times and associated secretarial workload, facilitating more structured follow-up (including data collection), and optimizing the patient care model (Figure S3 of the supplementary materials).

## 3. Discussion

To our knowledge, this is the first retrospective, observational study that describes clinical and economic outcomes of aboBoNT-A and onaBoNT-A treatments in toxin-naive adults with LS in a real-world setting. The redesigned service model in place at the North Staffordshire Rehabilitation Centre from March 2015 led to changes in practice for treatment of patients with LS, including the introduction of multidisciplinary assessments, and from 30 May 2017 a change of toxin used. These changes may have had a beneficial effect on clinical outcomes, patient satisfaction, and QoL. These findings are consistent with those of previously published studies recommending a multidisciplinary approach in the management of spasticity [3,5,7]. In addition, the improvements in clinical practice reported in this study may also be helpful in establishing an optimized patient pathway for spasticity management, representing a reconfiguration of existing healthcare resource to optimize service efficiencies

Although this study was exclusively retrospective without any *a priori* hypothesis to answer and was not powered to compare the two cohorts, we used exploratory analyses to describe the effect of toxin change on both clinical outcomes and treatment costs. Interestingly, the proportion of patients who achieved all therapeutic goals was numerically higher in Cohort 2, who were treated with aboBoNT-A, than in Cohort 1, who received onaBoNT-A. Furthermore, aboBoNT-A was associated with numerically higher treatment satisfaction and an improved QoL at Week 12 compared with onaBoNT-A. These results are similar to those from other studies that showed favorable outcomes for aboBoNT-A compared with onaBoNT-A [8,9,15,22,23,24].

Clinical outcomes at Week 6, as measured by mean GAS-T scores, for patients injected with aboBoNT-A (47.8) or onaBoNT-A (43.1), were broadly consistent with those reported in previously published studies [8,9,23,24]. The GAS-T score informs on the accuracy of goal setting. If set appropriately, the score should oscillate around 50, meaning that underachievement and overachievement of goals occur roughly equally. In this study, the mean GAS-T scores were below 50, suggesting that the treating teams may have set overambitious goals. Improving realistic goals setting with patients and accurately predicting BoNT-A treatment outcomes may be factors that will lead to the better use of Goal Attainment Scale (GAS) scores as an outcome measure at the North Staffordshire Rehabilitation Centre.

Overall, the safety profile of aboBoNT-A in this real-world setting was consistent with that in the summary of product characteristics [12], and was in agreement with that described in previously published studies that concluded that aboBoNT-A is a well-tolerated treatment for spasticity [23].

Although no statistical comparison was performed, the ad hoc analysis on estimated costs showed that treatment costs were numerically lower with aboBoNT-A than with onaBoNT-A at both Week 6 and Week 12. This is consistent with previous pharmacoeconomic studies that have suggested that switching from onaBoNT-A to aboBoNT-A could save costs [15,18]. For example, in one retrospective study comparing costs in 19 countries, the cost of aboBoNT-A in the management of LS was shown to be approximately 20% lower than that of onaBoNT-A per patient and per injection [15], which is similar in magnitude to the findings of the current study.

It should be noted that aboBoNT-A and onaBoNT-A are not chemically equivalent formulations. AboBoNT-A contains a greater amount of active neurotoxin when used at the recommended doses than onaBoNT-A, and the dose conversion ratios are still debated when patients switch from one to the other because aboBoNT-A and onaBoNT-A units are noninterchangeable [25,26]. One study that included patients with several types of dystonia suggested a dose conversion ratio of 1:4.61 (range, 2.3–9.6) when switching from onaBoNT-A to aboBoNT-A [10]. AboBoNT-A 900 U and onaBoNT-A 360 U doses (ratio 2.5:1) are undergoing head-to-head evaluation in the ongoing prospective DIRECTION study in adults with upper LS (ClinicalTrials.gov identifier, NCT04936542) [27].

The data from this single-center study suggest that aboBoNT-A is at least as effective as onaBoNT-A for the treatment of LS in routine clinical practice and has the potential to reduce treatment costs. Interestingly, aboBoNT-A has been shown to allow for longer time to retreatment in comparison with onaBoNT-A in patients with upper LS [8,9,26]. In the context of the COVID-19 pandemic, when countermeasures were implemented and routine visits delayed, use of a toxin that helps to lengthen the time between visits may be a step toward a positive change in practice. The data collected in this study may warrant a larger prospective study powered to compare the effectiveness, safety, and economic outcomes of aboBoNT-A and onaBoNT-A, and would need to include data on clinical rating of spasticity (e.g., using the Modified Ashworth Scale or Tardieu Scale).

### Limitations

The service redesign period spans the data collection period; therefore, no direct comparison can be made between cohorts. In addition, the data collected in this study are indirect measures of the effect of BoNT-A treatment and the study was not powered to compare onaBoNT-A and aboBoNT-A outcomes. To compare outcomes for these treatments, direct measures would require data on spasticity severity obtained using the Modified Ashworth Scale or Tardieu Scale at different time points. Furthermore, spasticity severity is used to determine treatment dosage, which in turn drives the effectiveness. Importantly, spasticity severity data were not collected in this study.

The authors acknowledge that the EQ-5D VAS might not be fully and specifically reflective of improvement in spasticity-related QoL; it is a widely used generic measure of overall well-being that has not been validated for use in spasticity [28].

As a single-center study, the results of the study may not be representative of the wider population of patients with spasticity treated with aboBoNT-A and/or onaBoNT-A in routine clinical practice across the UK. Given that this was an observational study, there was no randomization to treatment, and drug accountability information was not collected. We included all adults with a diagnosis of spasticity (except cerebral palsy). Despite the inclusive approach to patient eligibility, the sample size was relatively small, which made subgroup analyses (e.g., by specific diagnosis, or age) unfeasible. Similarly, the very small number of patients who switched BoNT-A preparation at reinjection (*n* = 4) precluded any comparative analysis of outcomes between the switch and non-switch subgroups.

The decision to prescribe aboBoNT-A or onaBoNT-A and other treatment decisions (including reinjections) were made before, and independently of, this study at the discretion of the treating physician and in line with the center’s routine clinical practice. This may have led to bias in the characteristics of patients selected for treatment as well as patient population heterogeneity. However, this potential bias was mitigated by the fact that all patients requiring toxin injections who met the eligibility criteria were considered for inclusion in this study, irrespective of clinical characteristics (no triaging). Additionally, there was continuity in practice during the collection period (same assessment teams and assessment formats), and GAS goals were set up and reviewed by the same two senior physiotherapists for the duration of the study. This consistency in goal setting and evaluation eliminated any potential differences over time that could have arisen from different perceptions of goal attainment, which is a previously reported limitation of GAS scoring. Furthermore, exact injection points in each muscle were not recorded. Several studies have shown that BoNT-A should be targeted based on the neural distribution rather than the muscle itself [29,30,31].

An additional limitation, which is inherent in retrospective, observational studies, is that the quality of the data used to evaluate the outcomes of interest was reliant on the accuracy and completeness of patients’ hospital medical records; furthermore, the number of patients with missing data varied for different endpoints. It was not possible to perform a preliminary data-quality assessment or to prospectively define the type and extent of data collected. Given that this was a retrospective study based on the secondary use of data previously collected for purposes other than research, not all confounding factors, bias, or errors could be accounted for or fully mitigated. This design also prohibited powering of the study to meaningfully compare the two cohorts directly via statistical analysis.

As a retrospective study evaluating longitudinal patient outcomes, hospital follow-up visits were scheduled according to routine clinical practice at that time. To maximize the data available for analysis of outcomes at follow-up visits, the actual visits were assigned to the most appropriate study week (Week 6, 12, or 24). Not all patients with a baseline visit had a Week 6, 12, or 24 visit. This is reflected in the presentation of data here (*n* values, missing data points). Moreover, visits were not all conducted face-to-face with the patients.

Another limitation is the fact that safety data were only collected for Cohort 2. It would be interesting to compare the safety data for aboBoNT-A and onaBoNT-A, and future studies should address this knowledge gap.

Finally, treatment cost analyses only included estimated BoNT-A treatment costs: changes in other health service costs were not measured.

## 4. Conclusions

The findings from this single-center UK study in a real-world setting of adults with LS suggest that a multidisciplinary approach and change in toxin may have a beneficial effect on clinical outcomes, patient satisfaction and QoL, and treatment costs. A larger head-to-head prospective clinical trial may be warranted in patients with LS to compare the clinical and economic outcomes of aboBoNT-A and onaBoNT-A treatments.

## 5. Materials and Methods

### 5.1. Study Design

This study (ClinicalTrials.gov identifier, NCT04396704) was a single-center, noninterventional, retrospective, longitudinal, real-world study conducted at the North Staffordshire Rehabilitation Centre, Haywood Community Hospital, Stoke-on-Trent, UK—part of the UK National Health Service (NHS) and managed by the Midlands Partnership NHS Foundation Trust. The study evaluated outcomes in patients who received onaBoNT-A treatment between 1 March 2015 and 29 May 2017 (Cohort 1) and, following a change in practice in 2017, in patients who received aboBoNT-A treatment between 30 May 2017 and 30 March 2019 (Cohort 2) (Figure S1).

Given that this was an observational study designed to reflect real-world clinical practice accurately, there was no randomization to treatment, and physicians were free to prescribe aboBoNT-A or onaBoNT-A, choose targeted muscles, injection characteristics (dose, frequency, volume), and methods of administration of the selected toxin in accordance with routine clinical practice at the North Staffordshire Rehabilitation Centre and the current local summaries of product characteristics. Physicians could use the following vials: onaBoNT-A 50 U, 100 U, or 200 U, or aboBoNT-A 300 U or 500 U.

Clinical outcomes were measured using GAS scores for ‘impairment and symptoms’ and ‘activities and function’. In addition, this study utilized patient-reported outcome measures for QoL and treatment satisfaction, as well as patient-level cost estimations.

### 5.2. Ethics

The study complied with the recommendations of the Declaration of Helsinki and EU Directive 95/46/EC of the European Parliament and of the Council of 24 October 1995 on the protection of individuals regarding the processing of personal data and on the free movement of such data. This study also followed the Guidelines for Good Pharmacoepidemiology Practices (GPP2), April 2007, from the International Society for Pharmacoepidemiology.

Before initiating the study, the North Staffordshire Rehabilitation Centre obtained written and dated approval or favorable opinion from the UK NHS Health Research Authority West Midlands—Edgbaston Research Ethics Committee (reference 20/WM/0203, 15 July 2020).

All data were collected by members of patients’ care teams using patients’ medical records and pseudonymized before transfer to external researchers. Given that patients’ medical records were only accessed by the care team and that patient confidentiality was preserved at all times during the study, patient consent was not required.

### 5.3. Inclusion and Exclusion Criteria

The study included adults with a diagnosis of spasticity related to any cause except cerebral palsy. Eligible patients had not received a BoNT-A injection during the 6 months before BoNT-A initiation and received either onaBoNT-A or aboBoNT-A during the observation period. Patients were treated at the North Staffordshire Rehabilitation Centre at BoNT-A initiation and throughout follow-up for up to 24 weeks after BoNT-A initiation.

Patients were excluded from entering the study if they were participating in an interventional clinical trial of an investigational medicinal product for the treatment of spasticity at index date and/or during follow-up.

### 5.4. Population

There was no pre-selection of patients based on clinical characteristics. For both treatment groups, all patients attending the clinic who required toxin injections and met the eligibility criteria were considered. Data were collected from all eligible patients during the eligibility periods. The sample size was driven by the number of patients who were initiated to receive treatment with aboBoNT-A or onaBoNT-A during the study eligibility periods. This study included patients who received at least one onaBoNT-A or aboBoNT-A injection cycle.

The full analysis set comprised all included patients: 60 patients treated with onaBoNT-A (Cohort 1) and 54 patients treated with aboBoNT-A (Cohort 2). The safety analysis set comprised all included patients in Cohort 2 at the index date (baseline). All effectiveness analyses were performed on the full analysis set. Safety analyses were performed on the safety analysis set.

### 5.5. Patient Follow-Up

Follow-up assessment visits were conducted as either a face-to-face review at the spasticity clinic or via a phone call from the spasticity clinic to the patient, patient’s caregiver, or patient’s treating healthcare professional. The planned study visit schedule is described in Figure S2. The index date was defined as the date of first BoNT-A injection for each patient, and follow-up was defined as the period starting at the index date lasting for up to 24 weeks after the index date or until reinjection (whichever was sooner). The observation period was defined as the baseline period (a maximum of 6 weeks before the index date) combined with the follow-up period (24 weeks after BoNT-A initiation). According to the protocol, the 24-week follow-up period had a strict data cut-off. In practice, investigators reported additional data after this cut-off, which are included in the observation period.

For the primary and secondary study objectives, treatment outcomes were evaluated at the index date and at patients’ subsequent routine assessments scheduled at Week 6 (±2 weeks) and Week 12 (±4 weeks) after the index date (Figure S2). Data including those from safety assessments and characteristics of follow-up and BoNT-A reinjection visits were also collected at the Week 24 visit, up to 24 weeks after the index date. The request for the Week 24 visit was initiated by the patient or their caregiver, and patients were given an appointment 2–4 weeks after the request.

### 5.6. Practice Changes and Continuity

The service redesign period spans the data collection period for this study and is detailed in Figure S3. Nonetheless, the continuity and consistency in practice during the collection period limited bias and allowed the authors to carry out this study. Firstly, all assessments, goal setting, and BoNT-A injections were carried out by fully experienced clinicians. For both cohorts, there was regular communication between the teams that made the assessments and those that collected the data. Each patient was assessed by a senior doctor and senior physiotherapist; although there was some variability, the principal investigator performed at least half of the assessments. The same physiotherapist carried out the initial assessment and the weeks 6 and 12 reviews for a specific patient. Secondly, the format of all the assessments (including patient-reported outcomes) remained the same throughout the study. In particular, GAS goals were set and reviewed by the same two senior physiotherapists for the duration of the observation period.

### 5.7. Study Objectives

#### 5.7.1. Primary Objective

The primary objective of this study was to describe the real-world clinical outcomes of practice change that included switching treatment from onaBoNT-A to aboBoNT-A in toxin-naive patients with LS, using GAS scores at Week 6 and Week 12 as outcome measures. To combine GAS scores on impairment and symptoms with those on activities and function (or the two goals on impairment and symptoms if no goal was set for activities and function) into a single measure, a GAS-T score was derived as a transformation of the GAS scores using an equal weight of 1 for each goal for all patients [32].

#### 5.7.2. Secondary Objectives

Secondary objectives included describing the injection characteristics (site of injection, mean total dose, mean dose per limb, and guided injection techniques, if recorded) at first BoNT-A injection and at reinjection, the proportion of patients who required reinjection by Week 12 (administered at the treating physician’s discretion in accordance with routine clinical practice at the North Staffordshire Rehabilitation Centre), and the time from first injection to reinjection during the 24-week follow-up. The timing of assessments was in line with usual site practice, which was to reinject within 24 weeks.

Other secondary objectives included describing patient-reported outcomes on QoL and treatment satisfaction at Week 6 and Week 12. General QoL was assessed using EQ-5D VAS, per routine practice at the center. The VAS records the patient’s self-rated health on a vertical 20 cm scale on which the endpoints are labeled “the best health you can imagine” (100) and “the worst health you can imagine” (0). This measure can be used as a quantitative measure of health as judged by each individual patient. Change in EQ-5D VAS from the baseline visit was calculated as the difference between baseline score and the score recorded at subsequent visits (Week 6 and Week 12). Patient satisfaction with treatment was determined at Week 6 and Week 12 using a Likert scale. Patients recorded their ‘best response’ (since index date) and ‘response now’ (at the time of the review) on a five-category scale: much worse, worse, the same, better, or much better.

Safety assessments were also evaluated for Cohort 2 (treated with aboBoNT-A) in the real-world setting.

### 5.8. Patient-Level Treatment Cost Estimations

Although no direct comparison could be made between the cohorts, an ad hoc analysis was performed in both cohorts to estimate the treatment costs for each patient at each visit. Vial prices were sourced in the British National Formulary (accessed via medicinescomplete.com, September 2021). Cost analyses followed the current clinical guidelines and summaries of product characteristics for aboBoNT-A and onaBoNT-A [9,10]. Treatment costs were based on the number of full vials used to cover the required dose for one patient or one injection only and vial unit price (analyses selected combinations of number and size of vials that provided the number of units closest to the dose injected).

At Week 6, cost calculations were based on both the first injection (baseline) and reinjection (for reinjected patients). At Week 12, they were based on the first injection and the reinjections at Week 6 and Week 12 (for reinjected patients). Costs per responder at Week 6 and Week 12 were estimated from treatment costs per patient, stratified by response.

### 5.9. Data Analysis

This study was purely retrospective and exploratory, and was not powered to compare the effectiveness of aboBoNT-A and onaBoNT-A. All descriptive analyses were undertaken in both Cohort 1 and Cohort 2 separately, except when otherwise stated. Descriptive data for GAS-T scores at each visit and for other endpoints are presented as arithmetic mean and median; 95% CI and SD; first quartile, third quartile, and range (minimum and maximum values); frequency count; or percentage, as appropriate for quantitative and qualitative variables.

Missing data were not imputed, except for dates. When dates were ambiguous because of missing days and/or months, standard imputation was applied: when a day was missing, the 15th day of the month was assumed; when both day and month were missing, the 1st day of July was assumed. If data were missing for any of the endpoints, the number of missing values was reported for each endpoint, but not included in the denominator as part of percentage calculations. Regarding missing GAS-T scores, when the number of goals assessed at a follow-up visit was not equal to the number of goals set at the baseline visit, the GAS-T score was not calculated and was analyzed as missing for the relevant visit.

### 5.10. Exploratory Analyses

This study was retrospective without any *a priori* hypothesis to answer and was not powered to compare the two cohorts. Nonetheless, exploratory statistical analyses and numerical comparisons were carried out after data collection to describe the potential effect of toxin change. Thus, the *p* values presented are to be interpreted in the exploratory sense only.

Numerical comparisons between cohorts were conducted for the proportion of patients who achieved all therapeutic goals, EQ-5D VAS scores, patient satisfaction, and estimated treatment costs.

Exploratory statistical analyses were conducted to compare GAS and GAS-T scores between the treatment groups at Week 6 and Week 12.

All statistical tests were two-sided. Differences in GAS-T scores between the cohorts were assessed using a Mann–Whitney U-test because data were not normally distributed. Differences in proportions were assessed for each endpoint and at each visit using Fisher’s exact test. To ensure that differences between aboBoNT-A and onaBoNT-A were not affected by other factors, multivariable linear regression models were produced for the primary endpoint relating to mean GAS-T scores at Week 6 and Week 12 visits separately.

## Figures and Tables

**Table 1:** Baseline demographics and clinical characteristics.

	Cohort 1 (Pre-2017) (*N* = 60)	Cohort 2 (Post-2017) (*N* = 54)
**Age at first BoNT-A injection**		
Mean (SD), years	58.4 (15.6)	57.7 (16.3)
Median (range), years	57.1 (23.0–85.2)	62.7 (18.2–90.5)
**Sex, *n* (%)**		
Male	26 (43.3)	22 (40.7)
Female	34 (56.7)	32 (59.3)
**Underlying neurological condition**		
Patients with data available, *n*	60	53
Stroke due to infarction, *n* (%)	18 (30.0)	17 (32.1)
Stroke–hemorrhagic, *n* (%)	2 (3.3)	8 (15.1)
Stroke–unspecified, *n* (%)	12 (20.0)	0 (0.0)
Chronic disease (MS and other), *n* (%)	15 (25.0)	12 (22.6)
Traumatic brain injury, *n* (%)	5 (8.3)	6 (11.3)
Other ^a^, *n* (%)	8 (13.3)	10 (18.9)
Not known, *n*	0	1
**Time from diagnosis of neurological condition to first BoNT-A injection**		
Patients with data available, *n*	41	40
Median (range), years	2.1 (0.3–29.7)	2.4 (0.1–27.0)
**Location of spasticity, *n* (%)**		
LL spasticity	30 (50.0)	30 (55.6)
UL spasticity	23 (38.3)	19 (35.2)
LL + UL spasticity	7 (11.7)	5 (9.3)
**Time from diagnosis of spasticity to first BoNT-A injection**		
Patients with data available, *n*	57	54
Median (range), years	0 (0.0–0.1)	0 (0.0–3.1)
**Baseline existing therapies for LS, *n* (%)**		
Prescribed antispasticity medication	28 (46.7)	25 (46.3)
Pain medication and opioid use	15 (25.0)	22 (40.7)
Physiotherapy and/or occupational therapy	35 (58.3)	28 (51.9)
No therapy recorded	12 (20.0)	10 (18.5)

^a^ This includes spinal cord injury, neurological infection, dystonia, and others (not specified). BoNT-A, botulinumtoxinA; LL, lower limb; LS, limb spasticity; MS, multiple sclerosis; SD, standard deviation; UL, upper limb.

**Table 2:** Characteristics of follow-up and BoNT-A reinjection visits.

	Cohort 1 (Pre-2017) (*N* = 60)		Cohort 2 (Post-2017) (*N* = 54)	
**Patients attending scheduled visit, *n* (%)**				
Week 6	42 (70.0)		40 (74.1)	
Week 12	40 (66.7)		26 (48.1)	
Week 24	22 (36.7)		22 (40.7)	
**Patients reinjected at each visit, *n* (%)**				
Week 6	0 (0.0)		0 (0.0)	
Week 12	5 (8.3)		7 (13.0)	
Week 24	11 (18.3)		15 (27.8)	
**Time between index date and reinjection**				
Patients reinjected during the 24-week follow-up ^a^, *n* (%)	16 (26.7)		22 (40.7)	
Mean (SD), weeks	16.4 (2.7)		17.4 (3.7)	
95% CI, weeks	15.1–17.7		15.9–19.0	
Median (Q1, Q3), weeks	16.1 (15.1, 17.3)		17.1 (15.1, 19.9)	
Patients reinjected during the observation period ^b^, *n* (%)	18 (30.0)		27 (50.0) ^c^	
Mean (SD), weeks	19.9 (9.1)		19.8 (6.2)	
95% CI, weeks	15.0–23.4		17.4–22.1	
Median (Q1, Q3), weeks	16.1 (15.4, 18.0)		18.1 (16.1, 23.1)	
**Limb injected at reinjection**				
Data available, *n*	14		31 ^c^	
LL only, *n* (%)	9 (64.3)		18 (58.1)	
UL only, *n* (%)	3 (21.4)		13 (41.9)	
LL + UL, *n* (%)	2 (14.3)		0 (0)	
**Total dose at reinjection (units in Cohort 1 and Cohort 2 are noninterchangeable)**	*n*	mean (SD), U	*n*	mean (SD), U
UL	5	214.0 (37.8)	13	696.2 (420.1)
LL	11	203.6 (64.3)	17 ^d^	708.8 (445.9)
UL and/or LL	14	236.4 (80.8)	30 ^c,d^	703.3 (427.5)

^a^ The 24-week follow-up period is defined as the period starting at the index date for up to 24 weeks after the index date; patients may have been lost to follow-up before 24 weeks if they were deceased or moved to a different center, or if their medical records became unavailable. ^b^ According to the protocol, the 24-week follow-up period had a strict data cut-off. In practice, investigators reported additional data after this cut-off, which are included in the observation period. Eleven patients attended a visit outside the 24-week follow-up period, seven of whom were reinjected (five with aboBoNT-A, two with onaBoNT-A). ^c^ Only 27 patients who were originally treated with aboBoNT-A were reinjected with such within the observation period, but four patients originally treated with onaBoNT-A switched to aboBoNT-A for reinjection. ^d^ One patient had missing data. AboBoNT-A, abobotulinumtoxinA; BoNT-A, botulinumtoxinA; CI, confidence interval; LL, lower limb; onaBoNT-A, onabotulinumtoxinA; Q1, first quartile; Q3, third quartile; SD, standard deviation; UL, upper limb.

**Table 3:** EQ-5D VAS scores with changes from baseline.

	Cohort 1 (Pre-2017) (*N* = 60)		Cohort 2 (Post-2017) (*N* = 54)	
**EQ-5D VAS score by visit ^a^**		*n*		*n*
Baseline, median (Q1, Q3)	54 (41.3, 70.0)	46	60 (50.0, 75.0)	35
Week 6, median (Q1, Q3)	55 (48.0, 75.0)	36	65 (50.0, 80.0)	24
Week 12, median (Q1, Q3)	65 (50.0, 75.0)	25	80 (62.5, 90.0)	15
**Change in EQ-5D VAS score ^a^**		*n*		*n*
Change from baseline to Week 6, median (Q1, Q3)	0 (−7.5, 0.0)	35	0 (−5.0, 0.0)	24
Change from baseline to Week 12, median (Q1, Q3)	0 (−10.0, 0.0)	24	−5 (−28.8, 0.0)	14

^a^ Change in EQ-5D VAS from the baseline visit was calculated as the difference between baseline score and the score recorded at subsequent visits. Q1, first quartile; Q3, third quartile; VAS, visual analog scale.

**Table 4:** BoNT-A treatment costs in real-life clinical management of toxin-naive adults with LS ^a^.

Costs (£)	Cohort 1 (Pre-2017) (*N* = 60)			Cohort 2 (Post-2017) (*N* = 54)		
	Mean (SD)	95% CI	*n*	Mean (SD)	95% CI	*n*
**Per patient**						
Week 6	315.56 (141.88)	279.66–351.46	60	249.25 (136.08)	212.96–285.55	54
Week 12	343.20 (189.54)	295.24–391.16	60	273.21 (159.09)	230.77–315.64	54
**Per responder**						
Week 6	276.40 (116.80)	246.85–305.95	15	233.80 (139.91)	198.40–269.20	22
Week 12	391.57 (268.97)	323.51–459.63	12	290.89 (182.94)	244.60–337.18	9

^a^ Treatment costs were estimated for each patient at each visit. At Week 6, costs were based on the first injection only (no patient was reinjected at Week 6); at Week 12, costs were based on both the first injection and reinjection (for patients reinjected at Week 12). Costs were derived from the number of vials required for each patient and each injection. The approach minimized costs, whereby analyses selected combinations of number and size of vials that provided the number of units closest to the dose injected (e.g., an injection of onaBoNT-A 300 U was costed with 1 × 200 U vial and 1 × 100 U vial, whereas an injection of onaBoNT-A 350 U was costed with 2 × 200 U vials). BoNT-A, botulinumtoxinA; CI, confidence interval; LS, limb spasticity; SD, standard deviation.

## Data Availability

Qualified researchers may request access to patient-level study data that underlie the results reported in this publication. Additional relevant study documents, including the clinical study report, study protocol with any amendments, annotated case report form, statistical analysis plan, and data set specifications, may also be made available. Patient-level data will be anonymized, and study documents will be redacted to protect the privacy of study participants. When applicable, data from eligible studies are available 6 months after the studied medicine and indication have been approved in the US and EU or after the primary manuscript describing the results has been accepted for publication, whichever is later. Further details on Ipsen’s sharing criteria, eligible studies, and process for sharing are available here: https://vivli.org/members/ourmembers/ (accessed on 13 December 2022). Any requests should be submitted to www.vivli.org (accessed on 13 December 2022) for assessment by an independent scientific review board.

## Supplementary Materials

The following supporting information is available online at https://www.mdpi.com/article/10.3390/toxins15090532/s1, Figure S1: Description of the overall study period, including the identification periods for onaBoNT-A and aboBoNT-A treatments; Figure S2: Observation period and description of study visits, including baseline and follow-up periods applied for both cohorts; Figure S3: Summary of service redesign at the North Staffordshire Rehabilitation Centre in the UK; Table S1: Administration sites and total first dose of BoNT-A by limb(s); Table S2: Distribution of muscles injected at first BoNT-A injection (baseline); Table S3: Localization methods used at first BoNT-A injection by site of administration; Table S4: Clinical outcomes of BoNT-A treatment in toxin-naive adults with LS; Table S5: SAE reporting in Cohort 2 (no safety data were collected for Cohort 1).
