# Supplementary material for: A UK Single-Center, Retrospective, Noninterventional Study of Clinical Outcomes and Costs of Two BotulinumtoxinA Treatments for Limb Spasticity
Source: Toxins (Basel). 2023 Aug 30;15(9):532. doi: 10.3390/toxins15090532 (PMC10534376; doi:10.3390/toxins15090532)
Supplement: Supplementary file 1 [file toxins-15-00532-s001.zip › toxins-2538802-supplementary.pdf]

toxins

MDPI

Supplementary Materials: A UK Single-Center, Retrospective, Noninterventional Study of Clinical Outcomes and Costs of Two BotulinumtoxinA Treatments for Limb Spasticity

Clive Bezzina, Vadim Degtiar, Natalya Danchenko, Pascal Maisonobe, Benjamin Davis, Emanuel Engmann, Elodie Guyon, Sophie Lecanuet and John Whalen

Table S1. Administration sites and total first dose of BoNT-A by limb(s) [a].

	Cohort 1 (pre-2017) (*N* = 60)		Cohort 2 (post-2017) (*N* = 54)	
**Limb injected at first injection**	*n* (%)		*n* (%)	
**LL only**	31 (51.7)		30 (55.6)	
**UL only**	24 (40.0)		20 (37.0)	
**LL + UL**	5 (8.3)		4 (7.4)	
**Total dose at first injection[b]**	*n*	mean (SD), U	*n*	mean (SD), U
**LL**	36	186.4 (97.9)	34	689.7 (449.9)
**UL**	29	194.8 (87.9)	24	718.8 (410.7)
**LL and/or UL**	60	206.0 (98.8)	54	753.7 (457.3)

[a] The maximum authorized dose of aboBoNT-A is as follows: UL, 1000 U; LL, 1500 U; UL + LL, 1500 U. The maximum authorized dose of onaBoNT-A is as follows: UL, 240 U; LL, 400 U; the maximum cumulative dose for multiple indications should not exceed 400 U in a 12-week interval.
aboBoNT-A, abobotulinumtoxinA; BoNT-A, botulinumtoxinA; LL, lower limb; onaBoNT-A, onabotulinumtoxinA; SD, standard deviation; UL, upper limb.
[b]Units in Cohort 1 and Cohort 2 are noninterchangeable units.

Table S2. Distribution of injected muscles at first BoNT-A injection (baseline), grouped my muscle category.

	Cohort 1 (pre-2017) (*N* = 60)	Cohort 2 (post-2017) (*N* = 54)
**Shoulder girdle, n**	1	2
Splenius capitis, n (%)	0 (0.0)	1 (1.9)
Sternocleidomastoideus, n (%)	0 (0.0)	1 (1.9)
Trapezius, n (%)	1 (1.7)	1 (1.9)
**Shoulder, n**	2	5
Pectoralis major, n (%)	2 (3.3)	5 (9.3)
Subscapularis, n (%)	1 (1.7)	1 (1.9)
**Arm, n**	17	12
Biceps brachii, n (%)	8 (13.3)	9 (16.7)
Brachialis, n (%)	13 (21.7)	12 (22.2)
**Forearm, n**	28	19

(Deep) flexor digitorum profundus, n (%)	10 (16.7)	11 (20.4)
Brachioradialis, n (%)	9 (15.0)	3 (5.6)
Extensor carpi ulnaris, n (%)	1 (1.7)	0 (0.0)
Extensor pollicis brevis, n (%)	1 (1.7)	0 (0.0)
Flexor carpi radialis, n (%)	10 (16.7)	6 (11.1)
Flexor carpi ulnaris, n (%)	11 (18.3)	5 (9.3)
Flexor digitorum superficialis, n (%)	19 (31.7)	19 (35.2)
Flexor pollicis longus, n (%)	5 (8.3)	6 (11.1)
**Hand, n**	3	3
Abductor pollicis brevis, n (%)	0 (0.0)	1 (1.9)
Adductor pollicis, n (%)	0 (0.0)	1 (1.9)
Flexor pollicis brevis, n (%)	0 (0.0)	3 (5.6)
Lumbricals, n (%)	3 (5.0)	0 (0.0)
**Hip, n**	8	5
Adductor brevis	1 (1.7)	1 (1.9)
Adductor longus	2 (3.3)	1 (1.9)
All functional adductors except gracilis and pectineus, n (%)	1 (1.7)	3 (5.6)
Iliopsoas, n (%)	4 (6.7)	1 (1.9)
Rectus femoris, n (%)	1 (1.7)	1 (1.9)
**Thigh, n**	11	11
Biceps femoris, n (%)	0 (0.0)	1 (1.9)
Gracilis, n (%)	5 (8.3)	5 (9.3)
Quadriceps femoris, n (%)	1 (1.7)	0 (0.0)
Semimembranosus, n (%)	10 (16.7)	11 (20.4)
**Leg and foot, n**	23	29
Extensor digitorum longus, n (%)	1 (1.7)	1 (1.9)
Extensor hallucis longus, n (%)	2 (3.3)	3 (5.6)
Flexor digitorum brevis, n (%)	2 (3.3)	0 (0.0)
Flexor digitorum longus, n (%)	2 (3.3)	2 (3.7)
Flexor hallucis brevis, n (%)	0 (0.0)	0 (0.0)
Flexor hallucis longus, n (%)	1 (1.7)	2 (3.7)
Tibialis anterior, n (%)	1 (1.7)	1 (1.9)
Tibialis posterior, n (%)	6 (10.0)	12 (22.2)
Triceps surae (gastrocnemius), n (%)	12 (20.0)	11 (20.4)
Triceps surae (soleus), n (%)	12 (20.0)	17 (31.5)

Categories are not mutually exclusive per patient
aboBoNT-A, abobotulinumtoxinA; BoNT-A, botulinumtoxinA; LL, lower limb; onaBoNT-A, onabotulinumtoxinA; UL, upper limb.

Table S3. Localization methods used at first BoNT-A injection by site of administration.

n (%) [a]	Cohort 1 (pre-2017) (*N* = 60)		Cohort 2 (post-2017) (*N* = 54)	
	**UL (*n* = 0) [b]**	**LL (*n* = 3) [b]**	**UL (*n* = 15)**	**LL (*n* = 24)**
**Ultrasound**	0 (0.0)	2 (66.7)	9 (60.0)	19 (79.2)
**Electrostimulation**	0 (0.0)	1 (33.3)	4 (26.7)	4 (16.7)
**Palpation**	0 (0.0)	0 (0.0)	2 (13.3)	3 (12.5)
**Electromyography**	0 (0.0)	0 (0.0)	1 (6.7)	1 (4.2)
**Missing data**	29	33	9	10

[a] Percentages based on the number of patients injected for whom data are available. One guidance method is expected per muscle; however, more than one could be used and the sum of the percentages may exceed 100%.

[b] No data were available on the localization method for the first UL injections for patients in Cohort 1. The localization method for the first LL injections was recorded for only three patients in this group. Routine practice in clinic was to use palpation/anatomical landmarks for injections proximal to the elbow (UL) or knee (LL), and electrostimulation for injections distal to the elbow (UL) or knee (LL). BoNT-A, botulinumtoxinA; LL, lower limb; UL, upper limb.

Table S4. Clinical outcomes of BoNT-A treatment in toxin-naive adults with LS.

	**Week 6**		**Week 12**	
	**Cohort 1 (pre-2017) (*N* = 60)**	**Cohort 2 (post-2017) (*N* = 54)**	**Cohort 1 (pre-2017) (*N* = 60)**	**Cohort 2 (post-2017) (*N* = 54)**
**GAS-T score**				
Patients who attended their visit, *n*	42	40	40	26
Patients with available data, *n*	40	39	36	22
Mean (SD)	43.1 (12.3)	47.8 (13.0)	43.1 (14.4)	44.3 (12.8)
95% CI	39.3–46.9	43.7–51.9	38.5–47.9	39.0–49.7
p value [a]	0.0694		0.7222	
**GAS goals**	*n* = 40	*n* = 39	*n* = 36	*n* = 22
**Achieved or overachieved all therapeutic goals**				
n	15	22	12	9
% (95% CI)	37.5 (22.5–52.5)	56.4 (40.8–72.0)	33.3 (17.9–48.7)	40.9 (20.4–61.5)
p value [b]	0.1165		0.5853	
**Exceeded all therapeutic goals**				
n	3	3	3	2
% (95% CI)	7.5 (0.0–15.7)	7.7 (0.0–16.1)	8.3 (0.0–17.4)	9.1 (0.0–21.1)
p value [b]	1		1	
**GAS-T score ≥ 50**				
n	17	24	15	9
% (95% CI)	42.5 (27.2–57.8)	61.5 (46.3–76.8)	41.7 (25.6–57.8)	40.9 (20.4–61.5]

p value [b]	0.1165	1

[a] Exploratory analysis of the difference between GAS-T scores in Cohort 1 and Cohort 2 (nonparametric Mann–Whitney U-test).
[b] Exploratory analysis of the difference in proportions of patients in Cohort 1 compared with Cohort 2 (Fisher's exact test).
BoNT-A, botulinumtoxinA; CI, confidence interval; GAS, Goal Attainment Scale; GAS-T, Goal Attainment Scale T score; LS, limb spasticity; SD, standard deviation.

Table S5. SAE reporting in Cohort 2 (no safety data were collected for Cohort 1).

**Overall summary of SAEs excluding special situations, *n* (%)** [a]	**Cohort 2 (post-2017) (*N* = 54)**
**Any SAE**	**8 (14.8)**
**Nervous system disorder**	**3 (5.6)**
Balance disorder	1 (1.9)
Parkinson's disease	2 (3.7)
Seizure	1 (1.9)
**Metabolism and nutrition disorders**	**1 (1.9)**
Dehydration	1 (1.9)
**Social circumstances**	**1 (1.9)**
Patient refusal of treatment	1 (1.9)
**General disorders and administration-site conditions**	**2 (3.7)**
Catheter-site pain	1 (1.9)
Treatment noncompliance	1 (1.9)
**Injury, poisoning, and procedural complications**	**2 (3.7)**
Fall	2 (3.7)
Femoral neck fracture	1 (1.9)
**Infections and infestations**	**3 (5.6)**
Gastroenteritis	1 (1.9)
Lower respiratory tract infection	1 (1.9)
Urinary tract infection	1 (1.9)
**Gastrointestinal disorders**	**1 (1.9)**
Constipation	1 (1.9)

[a] Percentages are based on the number of patients in the safety population. Categories are not mutually exclusive.
SAE, serious adverse event.

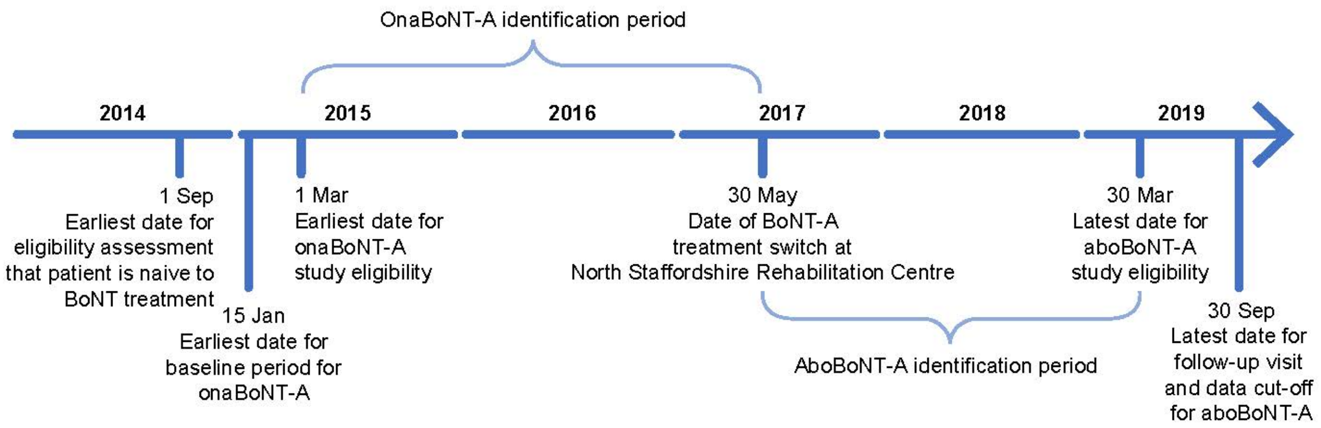

Figure S1. Description of the overall study period, including the identification periods for onaBoNT-A and aboBoNT-A treatments. AboBoNT-A, abobotulinumtoxinA, BoNT, botulinumtoxin; BoNT-A, botulinumtoxinA; onaBoNT-A, onabotulinumtoxinA.

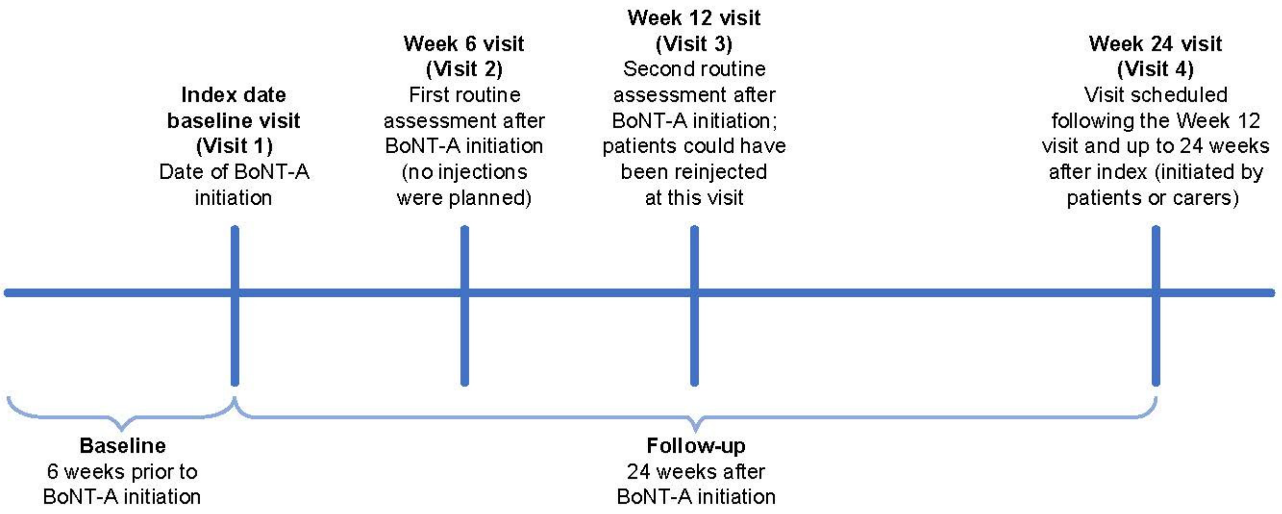

Figure S2. Observation period and description of study visits, including baseline and follow-up periods applied for both cohorts. The index date was defined as the date of first BoNT-A injection for each patient. Baseline measurements were defined as the observations recorded closest to, and no earlier than 6 weeks before, the first BoNT-A injection (the index event). It was expected that baseline measurements relevant to this study would have been made on the day of the first BoNT-A injection. Follow-up was defined as the period starting at the index date for up to 24 weeks after the index date; patients may have been lost to follow-up before 24 weeks if they were deceased, moved to a different center, or if their medical records became unavailable. Although the 24-week follow-up period had a strict data cut-off, the observation period included additional data reported by investigators after the 24-week cut-off because a number of patients attended a visit outside the 24-week follow-up period. BoNT-A, botulinumtoxinA.

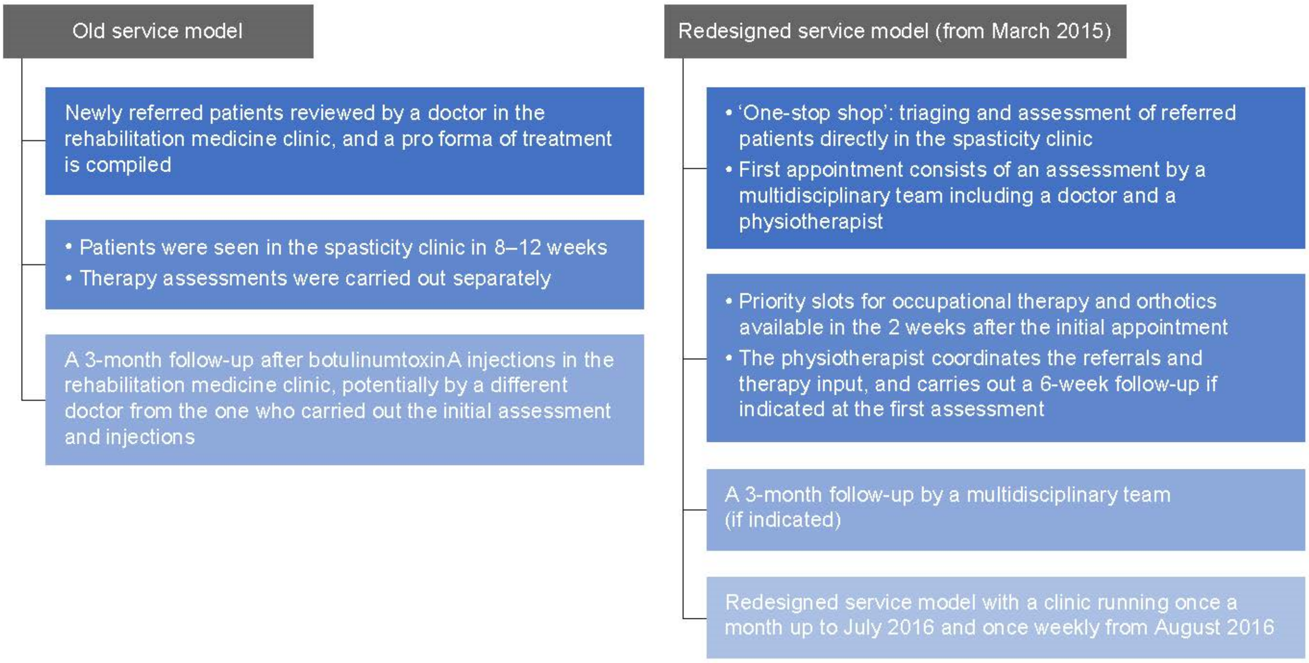

Figure S3. Summary of service redesign at the North Staffordshire Rehabilitation Centre in the UK.
